# Supplementary material for: ATG8 delipidation is not universally critical for autophagy in plants
Source: Nat Commun. 2025 Jan 5;16:403. doi: 10.1038/s41467-024-55754-1 (PMC11701075; doi:10.1038/s41467-024-55754-1)
Supplement: Supplementary file 1 — Supplementary Information [file 41467_2024_55754_MOESM1_ESM.pdf]

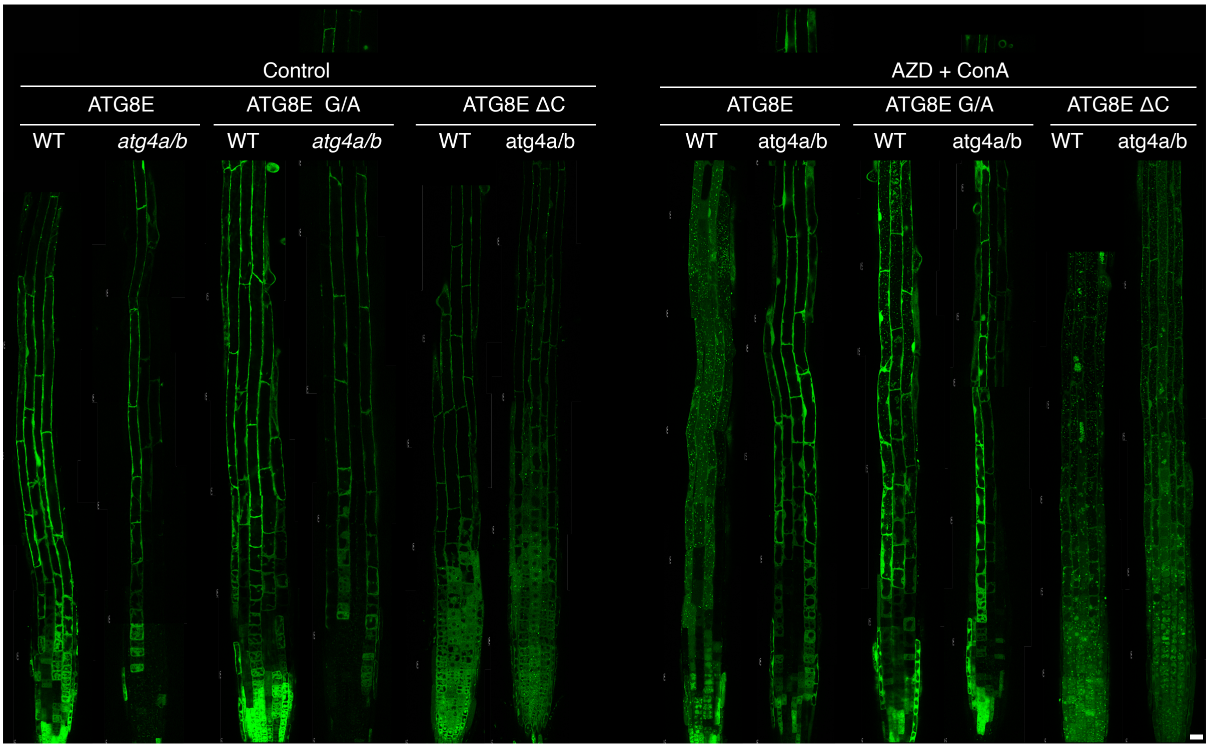

Supplementary Figure 1. **Expression of ATG8E ΔC restores accumulation of puncta in the vacuoles of ATG4-deficient Arabidopsis roots.**

Confocal microscopy images showing complete tile-scans of roots partially shown in **Fig. 1 D**. Control, roots incubated and imaged in the standard 0.5xMS growth medium; AZD + ConA, roots incubated and imaged in the 0.5xMS medium supplemented with 5 µM AZD8055 and 0.5 µM concanamycin A. Scale bar, 20 µm.

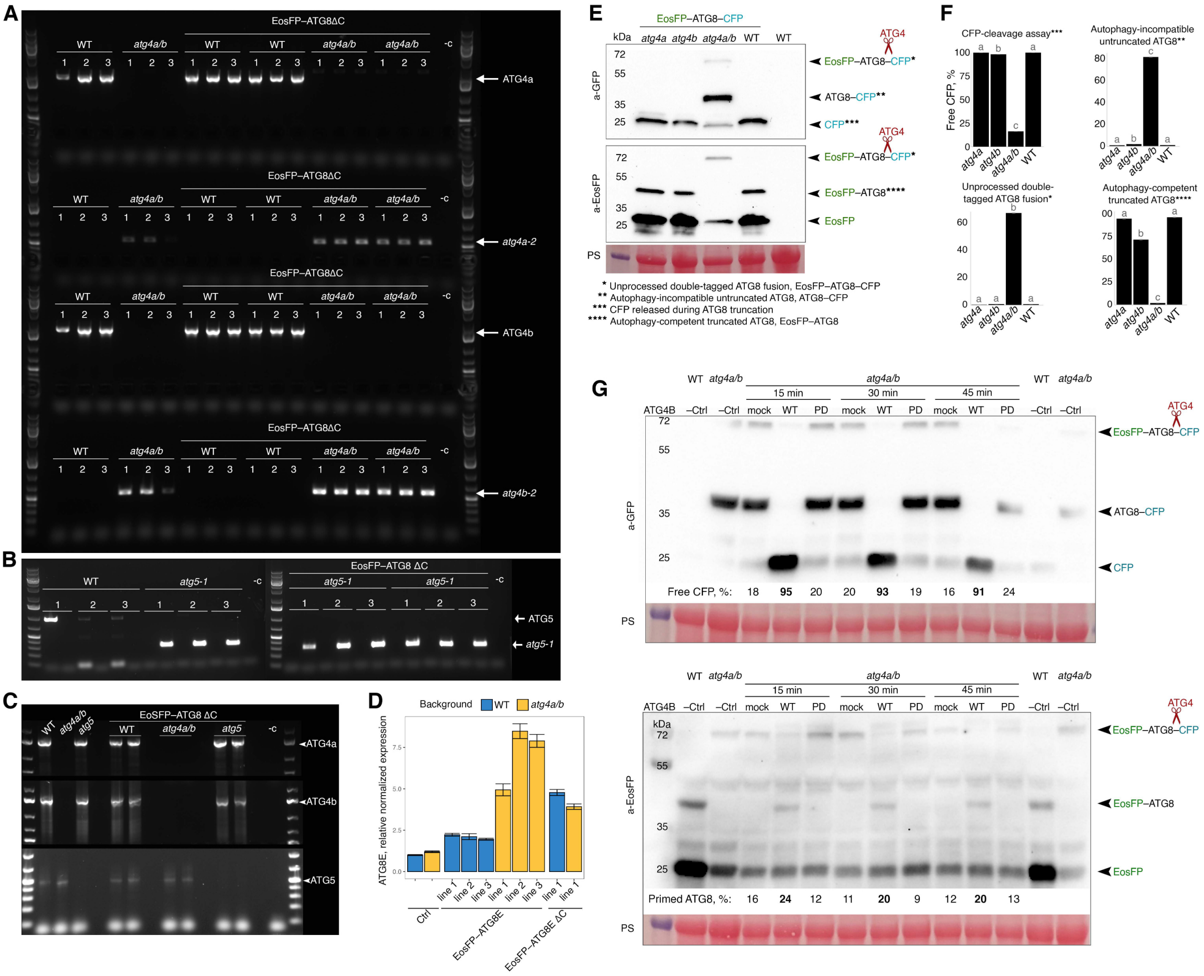

Supplementary Figure 2. Verification of the *atg4a/b* knockout and lines expressing EosFP-ATG8EΔC.

A. Genotyping confirms absence of intact *ATG4* alleles in all lines of the *atg4a/b* background.

B. Genotyping confirms absence of intact *ATG5* alleles in the *atg5-1* background lines.

C. RT-PCR confirms absence of full length *ATG4* mRNAs in the *atg4a/b* lines and ATG5 mRNA in *atg5-1* lines.

D. qPCR analysis of ATG8E expression in WT and *atg4a/b* lines with and without additional transgenes. Expression was normalized to the ATG8E level in WT and three reference genes.

E. *In planta* assay confirms lack of ATG4 activity in the *atg4a/b.* Processing of EosFP–ATG8–CFP expressed in the WT, *atg4a*, *atg4b,* and *atg4a/b* plants was detected in the total protein extracts. The product of cleavage, EosFP–ATG8, was not detectable in *atg4a/b*. Notably, expression of either of the ATG4 proteases is sufficient to process the overexpressed EosFP–ATG8–CFP. PS, Ponceau S.

F. Densitometric analysis of the Western blots in (**E**). Absence of both ATG4 proteases led to a drastic decrease in accumulation of free CFP (top left chart) and corresponding accumulation of the uncut EosFP–ATG8–CFP fusion (bottom left chart). Importantly, only in the absence of both ATG4 proteases, autophagy-incompatible ATG8–CFP was detectable (top right chart), while accumulation of autophagy-competent truncated EosFP–ATG8 form was dramatically decreased in the plants lacking both ATG4s. Tukey's HSD test, $n = 12$ plants.

G. *In vitro* assay corroborating ATG4-dependent processing of EosFP–ATG8–CFP. Total protein extracts of *atg4a/b* plants expressing EosFP–ATG8–CFP were incubated together with a buffer (mock), recombinant active ATG4B (WT) or its proteolytically dead mutant (PD) for the indicated time. Total protein extracts of plants expressing the same fusion protein in WT and *atg4a/b* were used as additional controls for fusion stability *in vivo* (WT –Ctrl and *atg4a/b* –Ctrl, respectively). Cleavage of the bond between ATG8 and CFP was observed only in the presence of active ATG4. Densitometry results represent integrated densities of the indicated bands expressed as % of the total signal intensity detected in the corresponding sample. PS, Ponceau S.

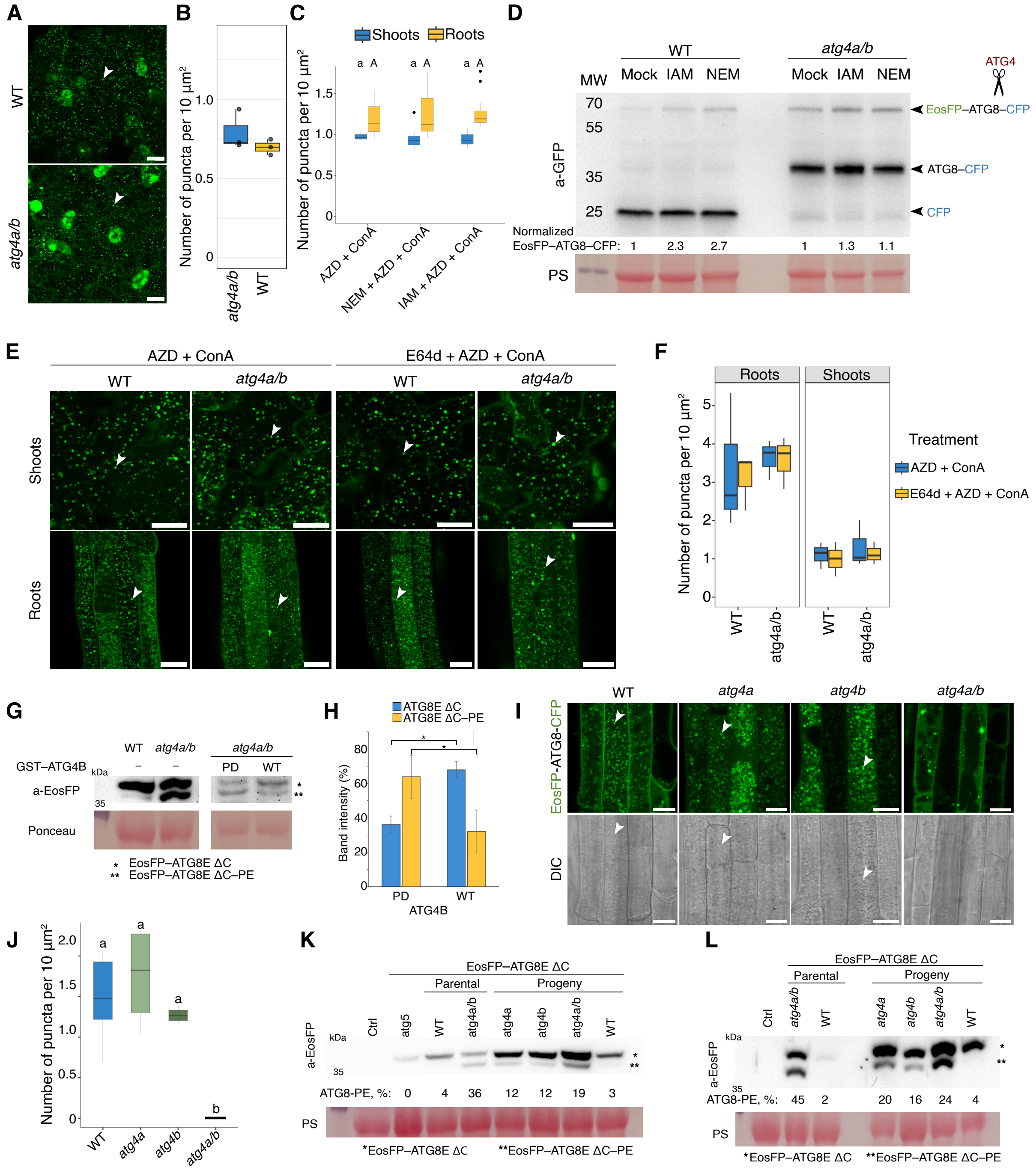

Supplementary Figure 3. ATG8 truncation is efficiently carried out by either ATG4 protease, but both are required for ATG8 delipidation.

A. CLSM of WT and *atg4a/b* Arabidopsis leaves expressing EosFP–ATG8EΔC treated with AZD/ConA.

B. Quantification of vacuolar puncta illustrated in (**A**). Data from one of two experiments, Welch's *t*-test, α = 0.05, *n* = 6 biological replicates (means as solid circles).

C. Quantification of vacuolar puncta exemplified in **Fig. 2C**. Data from one of two experiments. Tukey's HSD test, α = 0.05, n = 9 biological replicates.

D. Western blot showing accumulation of full-length EosFP–ATG8–CFP upon IAM or NEM treatment in WT, but not *atg4a/b* plants, confirming inhibition of ATG4 activity.

E. CLSM of WT or *atg4a/b* Arabidopsis seedlings expressing EosFP–ATG8EΔC treated with AZD/ConA with or without E64d.

F. Quantification of vacuolar puncta exampled in (**E**). Tukey's HSD test was performed for each organ, α = 0.05, *n* = 12 biological replicates per organ.

G. Western blot showing decrease in the ATG8–PE amounts upon incubation with recombinant active (WT) but not proteolytically dead mutant (PD) ATG4B. Assay performed on total protein extracts from *atg4a/b* and WT plants expressing EosFP–ATG8EΔC.

H. Densitometry of EosFP–ATG8EΔC and EosFP–ATG8EΔC–PE bands illustrated in **G,** expressed as % of total signal intensity for the corresponding sample. Results from two independent experiments. Student's *t*-test, $\alpha = 0.05$, *, $p < 0.001$, $n = 24$ plants.

I. CLSM of WT, *atg4a*, *atg4b* and *atg4a/b* Arabidopsis roots expressing EosFP–ATG8EΔC treated with AZD/ConA. Accumulation of autophagic bodies is inhibited only in the absence of both ATG4 isoforms. DIC, differential interference contrast.

J. Quantification of vacuolar puncta exampled in (**I**). Tukey's HSD test, $p < 0.05$, $n = 63$ seedlings.

K–L. Western blot of seedlings (**K**) and leaves (**L**) from WT, *atg4a*, *atg4b*, and *atg4a/b* plants expressing EosFP–ATG8EΔC, showing ATG8 delipidation requiring both ATG4A and ATG4B. –Ctrl, negative control.

Scale bars, 20 µm. White arrowheads indicate autophagic bodies. PS, Ponceau S. Numbers under Western blot lanes indicate densitometry of the uncleaved protein band as % of total signal intensity.

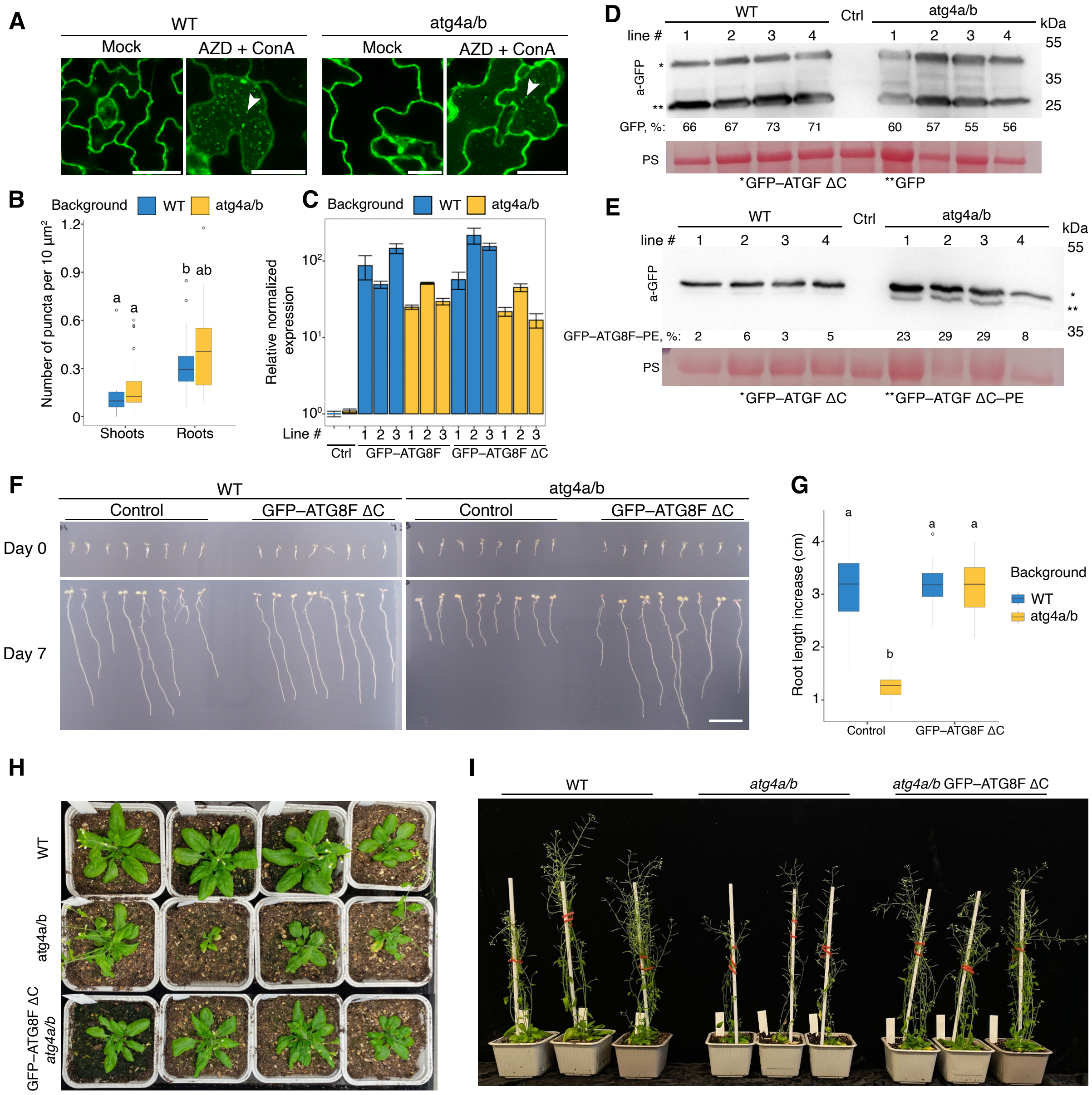

Supplementary Figure 4. Expression of artificially truncated ATG8F restores autophagic activity in ATG4-deficient background.

A. CLSM of WT and *atg4a/b* Arabidopsis plants expressing EosFP–ATG8FΔC in WT treated with AZD/ConA. White arrowheads indicate autophagic bodies. Scale bars, 20 µm.
B. Quantification of vacuolar puncta exampled in **A** and **Fig. 2I**. Representative data of one out of two experiments. Tukey's HSD test, $\alpha = 0.05$, $n = 20$ biological replicates.
C. qPCR analysis of ATG8F expression in WT and *atg4a/b* lines with and without additional transgenes. Expression was normalized to the ATG8F level detected in WT and to three reference genes. Error bars show SEM for the technical triplicates. Three individual lines were checked for each genotype.
D. GFP-cleavage assay confirms restored autophagic activity in *atg4a/b* plants expressing the GFP–ATG8FΔC and treated with AZD/ConA. Four individual lines were checked for WT and *atg4a/b* background.
E. Western blot detection of the lipidated ATG8 form. Protein extracts depicted in (**D**) were separated on a polyacrylamide gel containing 6 M urea to reveal GFP–ATG8FΔC–PE accumulation in the absence of ATG4 activity.
F. Expression of the artificially truncated ATG8FΔC isoform rescues the autophagy-deficient phenotype (stunted root growth on –N medium) of *atg4a/b*. Scale bar, 1 cm.
G. Quantification of root length increase in seedlings after 7 days of growth on the –N medium (illustrated in **F**). Representative data from one out of two experiments. Tukey's HSD test, $\alpha = 0.05$, $n = 47$ seedlings. Each seedling of WT or *atg4a/b* expressing GFP–ATG8FΔC represents an independent transgenic line.
H. Senescence onset is detectable in the rosette of 5-week-old *atg4a/b* plants, but not in WT or *atg4a/b* plants expressing ATG8FΔC. Each *atg4a/b* GFP–ATG8FΔC plant is an independent transgenic line. The experiment was repeated twice with four biological replicates per repetition.
I. Representative phenotypes of two-month-old plants, each *atg4a/b* GFP–ATG8FΔC plant is an individual transgenic line. The experiment was performed twice using four biological replicates for each repetition.

PS, Ponceau S. Numbers under Western blot lanes indicate densitometry of the band of interest expressed as % of total signal intensity in the sample.

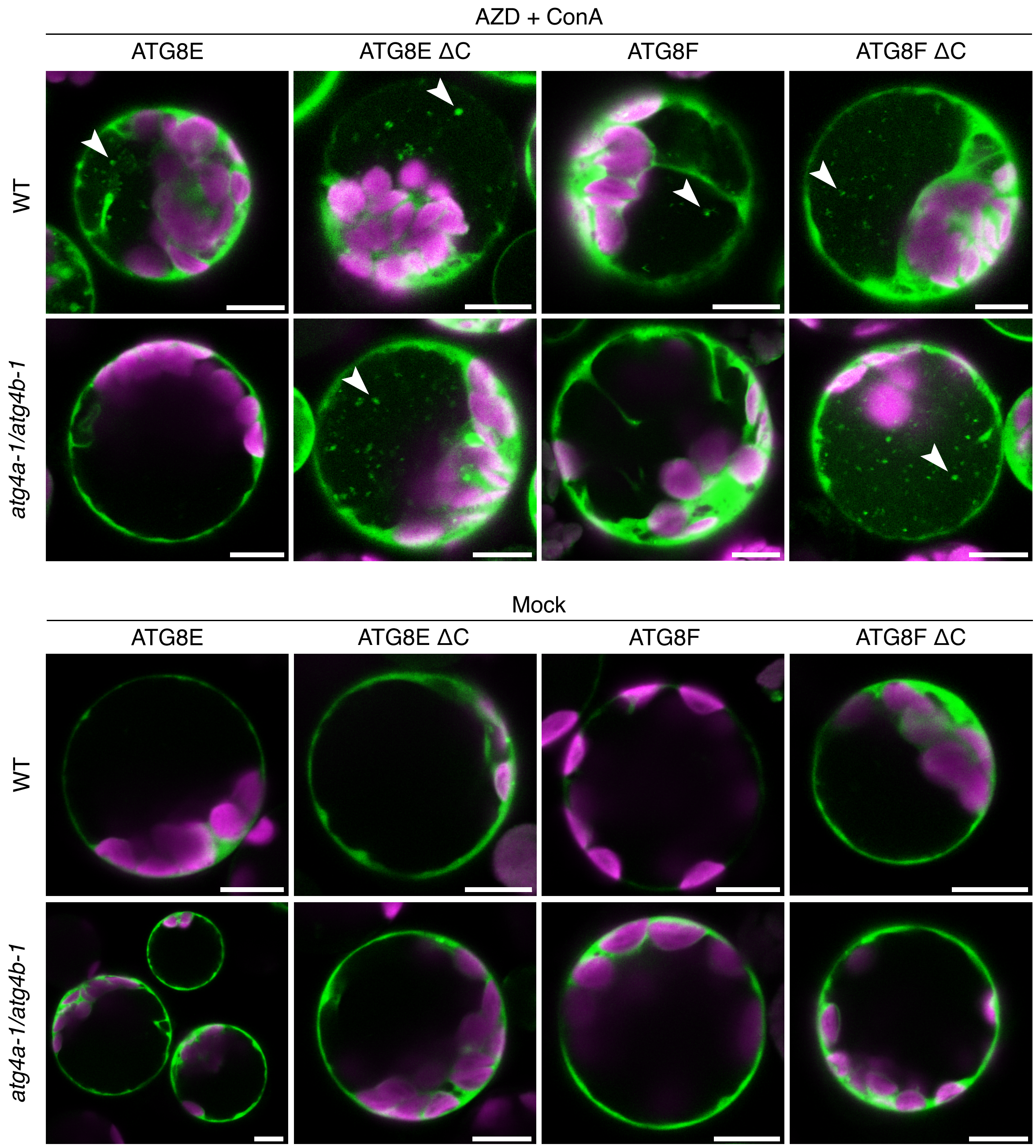

Supplementary Figure 5. Artificially truncated ATG8E and ATG8F restore accumulation of autophagic bodies in atg4a-1/b-1.

Confocal images of mesophyll protoplasts isolated from 3-week-old WT and *atg4a-1/4b-1* (both in Wassilewskija-0 ecotype) plants. Protoplasts were transformed to transiently express GFP fusions of full-length and artificially truncated versions of ATG8E and ATG8F isoforms. Protoplasts were either kept under standard conditions (mock) or subjected to 24 h treatment with AZD/ConA prior to imaging. White arrowheads point at autophagic bodies accumulating in the vacuoles of cells expressing truncated versions of ATG8E and ATG8F when treated with drugs to induce autophagy. Scale bars, 10 µm.

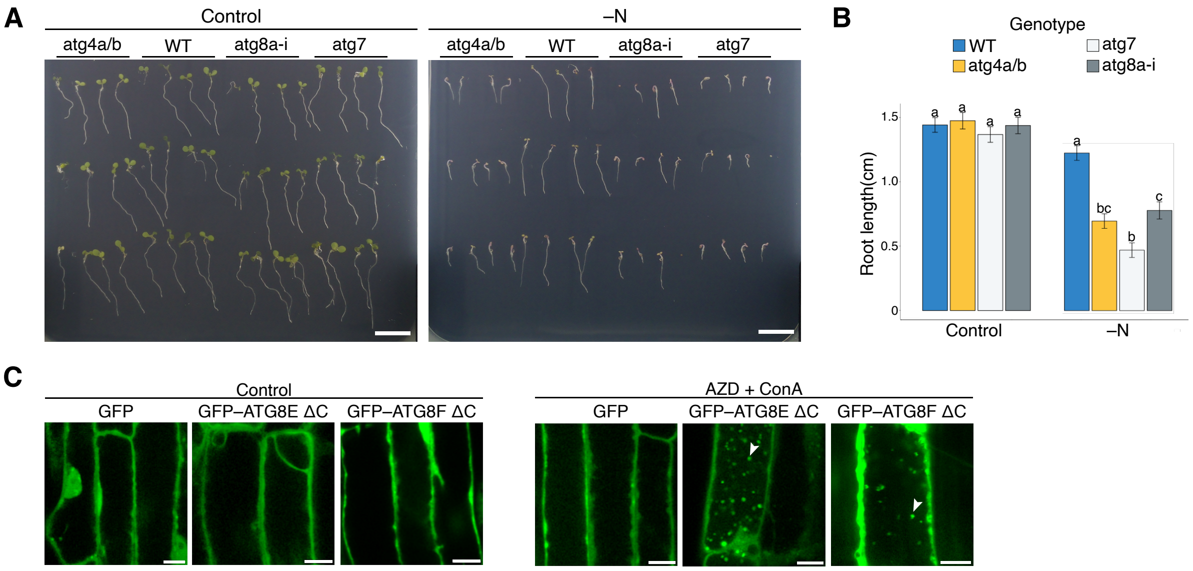

Supplementary Figure 6. Either of the artificially truncated ATG8F or ATG8E isoforms is sufficient to restore autophagic body accumulation in the absence of other ATG8 isoforms.

A. Autophagy-deficient phenotype of the nonuple ATG8-knockout line (*atg8a-i*) was assessed using SPIRO assay to track root growth on control and nitrogen-depleted media. Representative SPIRO image of 5-day-old seedlings on both types of media. Scale bar, 1 cm.

B. The SPIRO Root Growth assay results for the samples illustrated in (**A**) reveal stunted root growth in *atg8a-i* seedlings under nitrogen-deficient conditions, comparable to the phenotypes observed in other *ATG7*- and *ATG4*-knockout mutants. The chart shows predicted root lengths at 96 h after seed germination. Error bars represent SE. Distinct letters represent groups that are significantly different from each other, Tukey's HSD test, $\alpha = 0.05$. The chart shows representative results of one out of two experiments, $n = 218$ seedlings.

C. Confocal microscopy images of root epidermal cells. Prior to imaging, 7-day-old Arabidopsis seedlings expressing GFP–ATG8E ΔC, GFP–ATG8F ΔC or GFP in *atg8a-i* were treated for 2 h with 5µM AZD8055 and 0.5 µM ConA to induce autophagy or were kept under normal conditions (Control). White arrowheads point at autophagic bodies accumulating in the vacuoles. No autophagic bodies were observed in the vacuoles of seedlings expressing GFP. Scale bars, 20 µm.

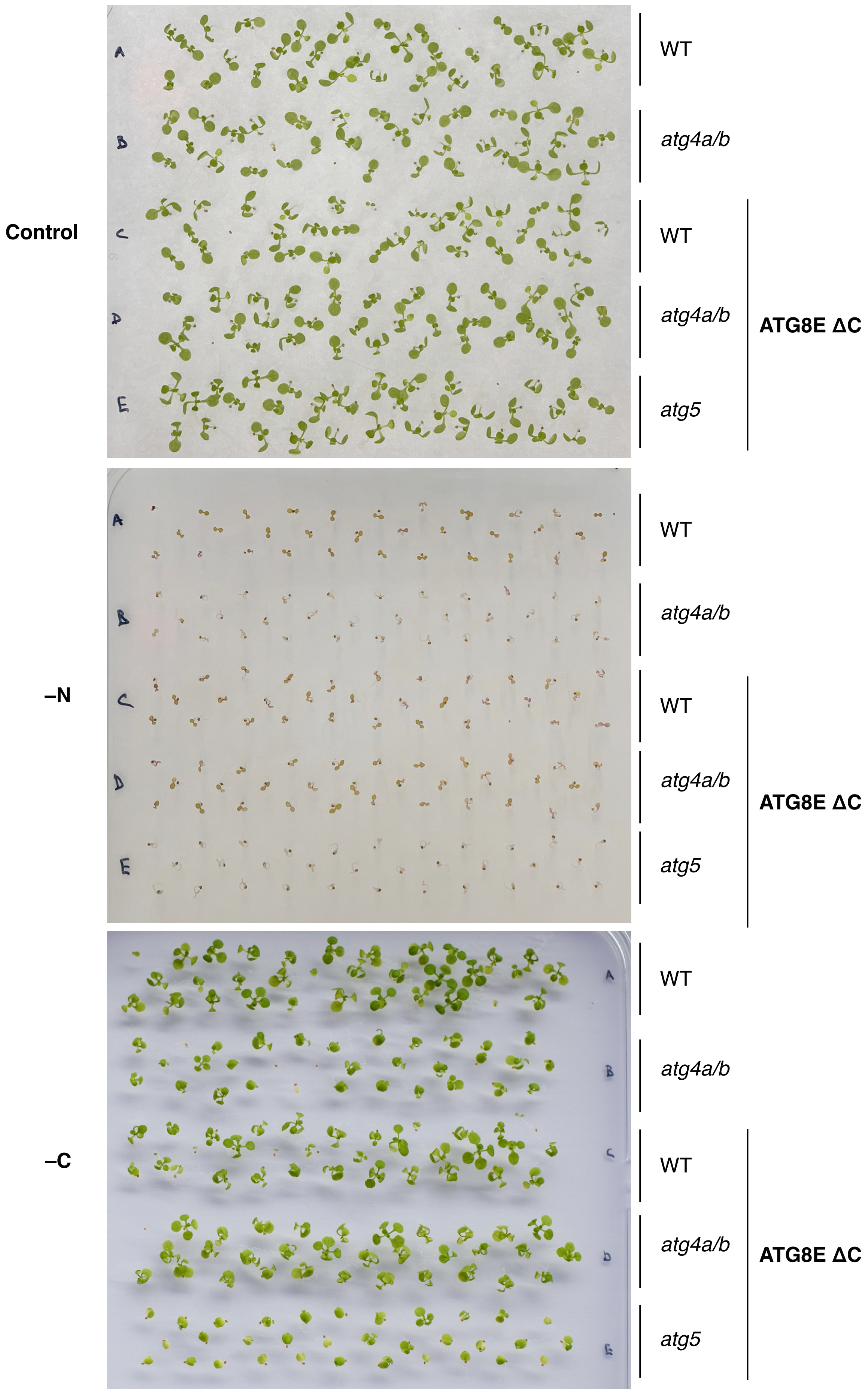
Control
WT
atg4a/b
WT
atg4a/b
atg5
ATG8E ΔC
–N
WT
atg4a/b
WT
atg4a/b
atg5
ATG8E ΔC
–C
WT
atg4a/b
WT
atg4a/b
atg5
ATG8E ΔC

Supplementary Figure 7. Expression of ATG8E ΔC complements autophagy-deficient shoot phenotypes of *atg4a/b*. Images of Petri plates with Arabidopsis seedlings grown on 0.5x MS medium (Control), nitrogen-depleted (–N) medium or carbon-depleted (–C) medium. Autophagy-deficient seedlings have decreased viability under –N conditions (quantification shown **Fig. 4E**). However, *atg4a/b* seedlings expressing ATG8E **Δ**C exhibit WT-like viability. Seedlings grown on –C medium were subjected to 7 days of dark treatment followed by seven days of recovery prior to imaging. Autophagy-deficient seedlings are slower to recover compared to both wild-type seedlings and *atg4a/b* seedlings expressing ATG8E **Δ**C (quantification shown in **Fig. 4F**).

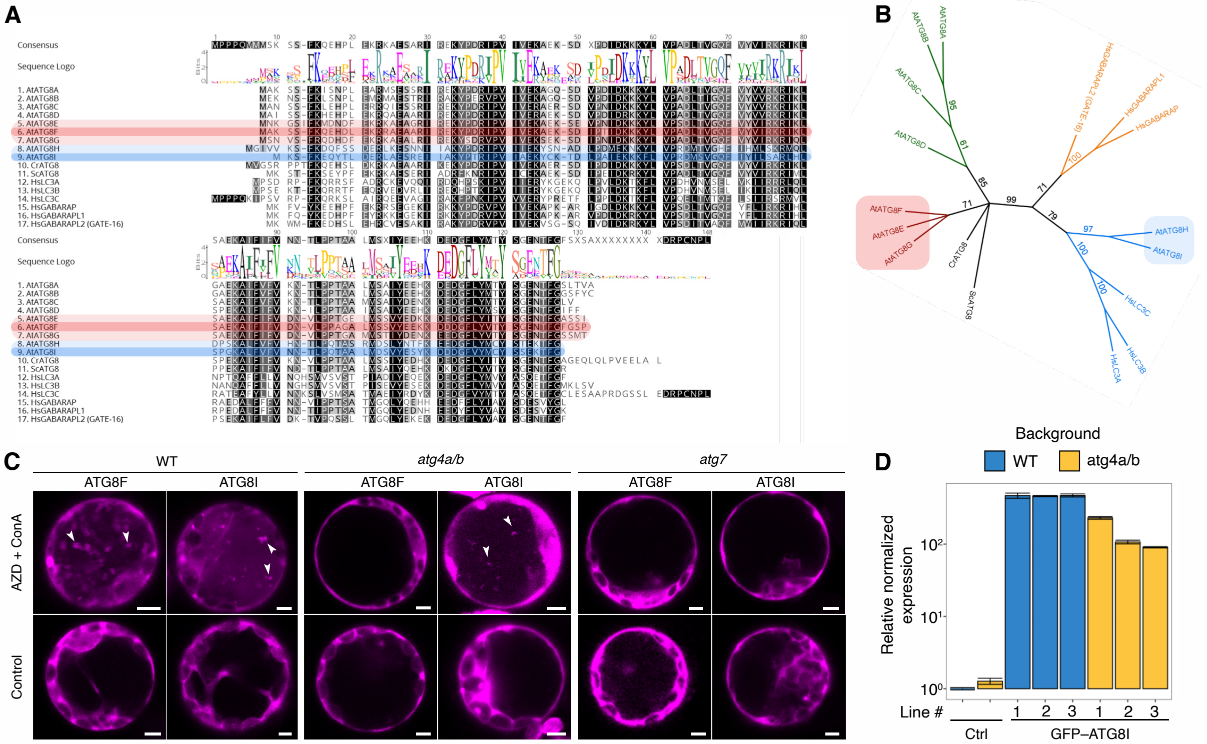

Supplementary Figure 8. Transient overexpression of the natively truncated ATG8I isoform restores accumulation of autophagic bodies in the vacuoles of *atg4a/b* cells.

A. ClustalW protein sequence alignment of *Arabidopsis thaliana* (At), *Chlamydomonas reinhardtii* (Cr), *Saccharomyces cerevisiae* (Sc) and *Homo sapiens* (Hs) orthologs of ATG8. Sequences of complete and truncated versions of Arabidopsis ATG8s with the highest expression levels, ATG8F and ATG8I, are highlighted in red and blue, respectively. Sequences of their closest paralogs are highlighted in lighter shades of the same colors.

B. Phylogenetic tree illustrating evolutionary distances of ATG8 proteins compared in **A**, shows the separation of Arabidopsis ATG8s into the previously described two clades[14]: Clade I, more similar to fungi and comprising only complete ATG8s containing the C-terminus, and Clade II, comprising natively truncated ATG8s, more similar to animal ATG8s.

C. mScarlet–ATG8F or mScarlet–ATG8I were transiently expressed in mesophyll protoplasts of WT, *atg4a/b,* or *atg7* Arabidopsis plants. An aliquot of each transformed protoplast suspension was subjected to AZD/ConA treatment for 17 h prior to imaging. Scale bars, 5 µm. White arrowheads indicate autophagic bodies.

D. qPCR analysis of ATG8I expression in WT and *atg4a/b* lines with and without additional transgenes. Expression was normalized to the ATG8I level detected in WT and to three reference genes. Error bars show SEM for the technical triplicates. Three individual lines were checked for each genotype.

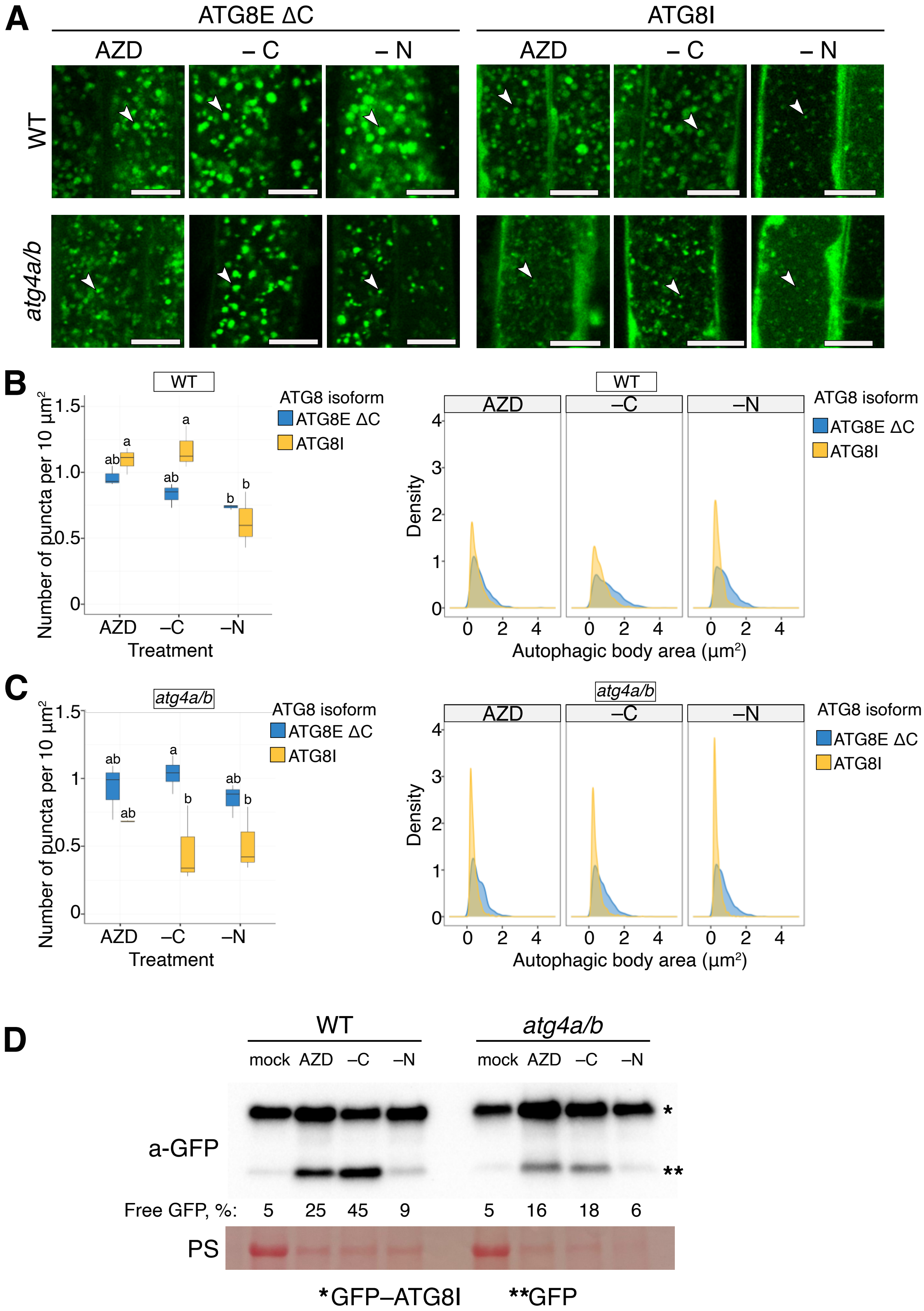

Supplementary Figure 9. Overexpression of the natively truncated ATG8I isoform in WT leads to decreased autophagic activity under nitrogen-depleted conditions.

A. CLSM of Arabidopsis roots expressing ATG8EΔC or ATG8I in WT and *atg4a/b* backgrounds treated with AZD/ConA, –C/ConA, and –N/ConA. Scale bars, 10 µm. White arrowheads indicate autophagic bodies of variable sizes.

B. Vacuolar puncta quantification (left, box plot) and their size (right, density plot) for the WT seedlings illustrated in (**A**). Overexpression of ATG8I in the WT background led to a decreased number of autophagic bodies only under nitrogen-depleted conditions. Furthermore, ATG8I-overexpressing WT seedlings showed the most prominent decrease in autophagic body size under –N conditions. Significance of differences in puncta number was assessed with Tukey's HSD test ($\alpha = 0.05$), $n = 18$ biological replicates (510 technical replicates). The kernel density plot shows probability density function for autophagic body size depending on the expressed ATG8 isoform and treatment, $n = 8001$ vacuolar puncta.
C. Quantification analogous to the one shown in (**B**) performed for the *atg4a/b* seedlings. Tukey's HSD test, $n = 18$ biological replicates (530 technical replicates). Kernel density plot, $n = 9153$ vacuolar puncta. Overexpression of ATG8I in the *atg4a/b* background revealed lower number of autophagic bodies under all three conditions, indicative of the ATG8I isoform being insufficient for complete restoration of autophagic flux in the absence of other ATG8 isoforms. In agreement with this observation, ATG8I-overexpressing *atga4a/b* seedlings exhibited a drastic decrease in autophagic body size under nitrogen-inducing conditions.
D. GFP-cleavage assay performed on the seedlings overexpressing GFP–ATG8I and illustrated in (**A**). The Western blot confirms autophagic activity across all three stress conditions employed in the experiment, validating that nitrogen depletion produces the lowest autophagic flux when GFP–ATG8I is overexpressed and supporting our observation that ATG8I fails to restore normal autophagic activity in *atg4a/b* pants. Densitometry results are shown as numbers under corresponding lanes and represent integrated density for the free GFP protein band expressed as % of total signal detected in the corresponding sample. PS, Ponceau S.

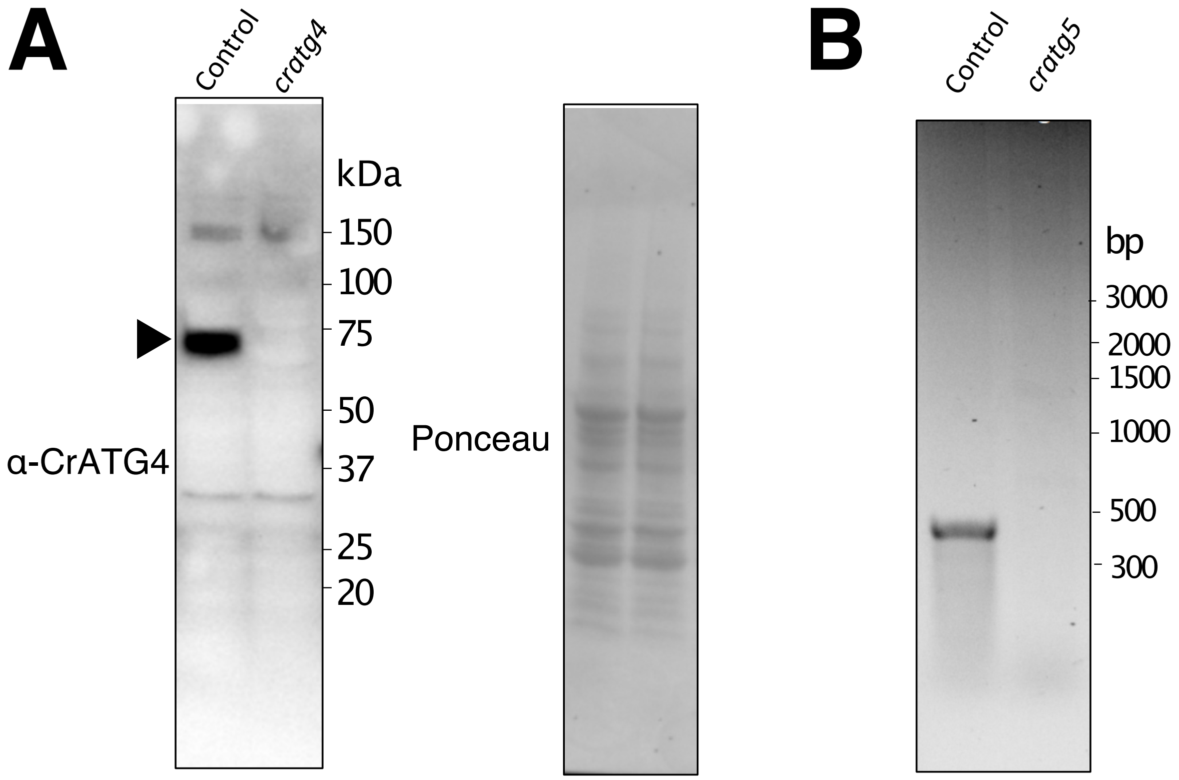

Supplementary Figure 10. Validation of generated *Chlamydomonas ATG4* and *ATG5* knockout mutants.
A. Western blot detection of ATG4 protein in the total protein extracts of WT (Control) and ATG4 knockout (cratg4) Chlamydomonas cells confirms absence of ATG4 protein in the knockout background. Ponceau S staining was used as a loading control.
B. Genotyping of the ATG5 knockout mutant confirms deletion of the ATG5 gene

Supplementary Table 1. List of primers used in this study.

		Arabidopsis	
**Primer no.**	**Primer name**	**5' → 3' sequence**	**Application**
AM 409	ATG4a.Fw	ATGAAGGCTTTATGTGATAGATTTGTTC	Arabidopsis genotyping
AM 410	ATG4a.Rev	TCAGAGCATTTGCCAGTCATCTTCAC	Arabidopsis genotyping
AM 420	attB2 ATG4b	GGGGACCACTTTGTACAAGAAAGCTGGGTAAAGTAATTGCCAGTCATCTT	Arabidopsis genotyping
AM 584	LBa1 (SALK)	TGGTTCACGTAGTGGGCCATCG	Arabidopsis genotyping
AM 587	ATG4a gen Re2	TGCAATGATAGGAGGATGTGTC	Arabidopsis genotyping
AM 417	attB1 ATG4a	GGGGACAAGTTTGTACAAAAAAGCAGGCTTAATGAAGGCTTTATGTGATAGA	Creating Gateway-compatible entry clone
AM 418	attB2 ATG4a	GGGGACCACTTTGTACAAGAAAGCTGGGTAGAGCATTTGCCAGTCATC	Creating Gateway-compatible entry clone
AM 419	attB1 ATG4b	GGGGACAAGTTTGTACAAAAAAGCAGGCTTAATGAAGGCTATATGTGATAGAT	Creating Gateway-compatible entry clone
AM 420	attB2 ATG4b	GGGGACCACTTTGTACAAGAAAGCTGGGTAAAGTAATTGCCAGTCATCTT	Creating Gateway-compatible entry clone
AM 475	attB1 AtATG8e gene	GGGGACAAGTTTGTACAAAAAAGCAGGCTTAATGAATAAAGGAAGCATCTTTAA	Creating Gateway-compatible entry clone
AM 476	attB2 AtATG8e gene no stop	GGGGACCACTTTGTACAAGAAAGCTGGGTAGATTGAAGAAGCACCGAATG	Creating Gateway-compatible entry clone
AM 477	attB2 AtATG8e gene Gly stop	GGGGACCACTTTGTACAAGAAAGCTGGGTATTAACCGAATGTGTTCTCGCCACT	Creating Gateway-compatible entry clone
AM 478	attB2 AtATG8e gene Gly/Ala	GGGGACCACTTTGTACAAGAAAGCTGGGTAGATTGAAGAAGCAGCGAATG	Creating Gateway-compatible entry clone
AM 611	pGGC AtATG8e Fw	AACAGGTCTCAGGCTCAACAATGAATAAAGGAA	Creating GreenGate-compatible entry clone
AM 643	pGGC AtATG8s delta C Re	AACAGGTCTCTCTGATTACCCAAAAGTGTTCTCTCCACTGTAAGT	Creating GreenGate-compatible entry clone
SH PR 34	35S.OH_FW	AACAGGTCTCAACCTCCTGCAGGTCAACATGGTGGAGCAC	Creating GreenGate-compatible entry clone
SH PR 35	35S.OH_Rev	AACAGGTCTCTTGTTTCCTCTCCAAATGAAATGAACTTCCT	Creating GreenGate-compatible entry clone
SH PR 10	pGGC_AtATG8i_Rev	AACAGGTCTCTCTGATCAACCAAAGGTTTTCTCACTGC	Creating GreenGate-compatible entry clone
SH PR 7	pGGC_AtATG8f_Fw	AACAGGTCTCAGGCTCAACAATGGCAAAAAGCTCGTTC	Creating GreenGate-compatible entry clone
SH PR 8	pGGC_AtATG8f_Rev	AACAGGTCTCTCTGATTATGGAGATCCAAATCCAAATGT	Creating GreenGate-compatible entry clone
SH PR 9	pGGC_AtATG8i_Fw	AACAGGTCTCAGGCTCAACAATGAAATCGTTCAAGGAACAATACA	Creating GreenGate-compatible entry clone
AM 427	CFP.dt.Fw	TCTCTTACCGTTGCTATGGTGAGCAAGGGC	Overlay PCR for double-tagged ATG8A
AM 428	attB2.CFP.dt.Re	GGGGACCACTTTGTACAAGAAAGCTGGGTACTACTTGTACAGCTCGTCCATGCCG	Overlay PCR for double-tagged ATG8A
AM 429	AtATG8a.dt.Fw	GGGGCACAAACTTAATATGATCTTTGCTTGCT	Overlay PCR for double-tagged ATG8A

AM 430	AtATG8a.dt.Re	GCCCTTGCTCACCATAGCAACGGTAAGAGA	Overlay PCR for double-tagged ATG8A
AM 431	attB1.AtATG8a.new	GGGGACAAGTTTGTACAAAAAAGCAGGCTTAATGATCTTTGCTTGCTTGAAATTC	Overlay PCR for double-tagged ATG8A
AM 225	Hel qPCR Fw	CCATTCTACTTTTTGGCGGCT	qPCR
AM 226	Hel qPCR Re	TCAATGGTAACTGATCCACTCTGATG	qPCR
AM 227	PP2A-qPCR-Fw	TAACGTGGCCAAAATGATGC	qPCR
AM 228	PP2A-qPCR-Rv	GTTCTCCACAACCGCTTGGT	qPCR
AM 229	UBC9-qPCR-Fw	TCACAATTTCCAAGGTGCTGC	qPCR
AM 230	UBC9-qPCR-Rv	TCATCTGGGTTTGGATCCGT	qPCR
AM 502	qPCR.ATG8E.Fw	TCTTCCTCCAACAGGAGAGC	qPCR
AM 503	qPCR.ATG8E.Re	CGAATGTGTTCTCGCCACTG	qPCR
AM 504	qPCR.ATG8F.Fw	TCCTGATAGGATTCCGGTGATTGTT	qPCR
AM 505	qPCR.ATG8F.Re	AGTCAGATCAGCCGGGACT	qPCR
AM 506	qPCR.ATG8I.Fw	GGATCTGCCTGCCATCGAG	qPCR
AM 507	qPCR.ATG8I.Re	CAGAGCAGCAGTTTGAGGG	qPCR
AM 622	ATG4a C/A Re	GGCTACTTCGAATCATGGCACCCCAGTTCACATCGC	Site-directed mutagenesis
AM 623	ATG4a C/A Fw	GCGATGTGAACTGGGGTGCCATGATTCGAAGTAGCC	Site-directed mutagenesis
AM 624	ATG4b C/A Fw	GGCCGCTTCGAAGCATGGCACCCCAGTTTACATCAC	Site-directed mutagenesis
AM 625	ATG4b C/A Re	GTGATGTAAACTGGGGTGCCATGCTTCGAAGCGGCC	Site-directed mutagenesis

Chlamydomonas

**Primer name**	**5' → 3' sequence**	**Application**
AphVIII-F	CTGTGGGTCTCATGCCGAAT	Cloning of AphVIII as the DNA donor
AphVIII-R	AGCTTGAATTCTTGCAGCGC	Cloning of AphVIII as the DNA donor
CrATG4-gRNA	/AltR1/rArArGrUrGrCrArCrCrArCrArUrUrCrGrUrGrArGrGr UrUrUrUrArGrArGrCrUrArUrGrCrU/AltR2/	crRNA for *CrATG4* gene
CrATG5-gRNA	/AltR1/rCrUrGrUrArCrGrArCrCrUrGrCrUrUrUrCrArGrArGr UrUrUrUrArGrArGrCrUrArUrGrCrU/AltR2/	crRNA for *CrATG5* gene
CrATG4-F	TTCGAGTGGCCACCTTCAAA	PCR detection of *CrATG4*
CrATG4-R	ACAACAACCCTAGCTGCTCA	PCR detection of *CrATG4*

CrATG5-F	ATCGCATTGTGCACAAACCC	PCR detection of *CrATG5*
CrATG5-R	GGGTAGGTGGAGCAGGTAGA	PCR detection of *CrATG5*
CrATG8-F	TTGAAGACAATTCGATGGTTGGCTCCCGACCC	Cloning of CrATG8 gene
CrATG8-R	TTGAAGACAAAAGCTCACAACGCCAGTTCCTCC	Cloning of CrATG8 gene
CrATG8ΔC -R	TTGAAGACAAAAGCTCAACCGAACGTGTTCTCGCC	Cloning of CrATG8ΔC gene without C terminal (with CrATG8-F)

Supplementary Table 2. Plasmids used in this study.

		Arabidopsis			
			Selection marker in		
**Construct no.**	**Construct name**	**Insert**	**plants**	**bacteria**	**Vector type**
**AM 779**	pGGZ 2x35S::E2GFP-AtATG8E	GFP-ATG8E CDS	FastRed	spec	Expression in plants
**AM 556**	pDONR/Zeo AtATG8A gene-CFP	AtATG8A gene-CFP	–	zeo	Entry clone
**AM 567**	pUBN ubi10::EOSFP-AtATG8A gene-CFP	AtATG8A gene-CFP	basta	spec	Expression in plants
**AM 655**	pDONR/Zeo AtATG8E gene	AtATG8E gene	–	zeo	Entry clone for Gateway cloning
**AM 656**	pDONR/Zeo AtATG8E gene Gly stop	AtATG8E gene Gly stop	–	zeo	Entry clone for Gateway cloning
**AM 657**	pDONR/Zeo AtATG8E gene Gly/Ala	AtATG8E gene Gly/Ala	–	zeo	Entry clone for Gateway cloning
**AM 661**	pUBN ubi10::EOSFP-AtATG8E gene no stop	AtATG8E gene no stop	basta	spec	Expression in plants
**AM 662**	pUBN ubi10::EOSFP-AtATG8E gene Gly stop	AtATG8E gene Gly stop (ATG8E ΔC)	basta	spec	Expression in plants
**AM 663**	pUBN ubi10::EOSFP-AtATG8E gene Gly/Ala	AtATG8E gene Gly/Ala	basta	spec	Expression in plants
**AM 753**	pDONR/Zeo AtATG4A CDS	AtATG4A CDS	–	zeo	Entry clone for Gateway cloning
**AM 754**	pDONR/Zeo AtATG4B CDS	AtATG4B CDS	–	zeo	Entry clone for Gateway cloning
**AM 755**	pDONR AtATG4A C/A	AtATG4A CDS C/A mutant	–	zeo	Entry clone for Gateway cloning
**AM 760**	pDONR AtATG4B C/A	AtATG4B CDS C/A mutant	–	zeo	Entry clone for Gateway cloning
**AM 780**	pGGZ 2x35S::E2GFP-AtATG8F	GFP-ATG8F CDS	FastRed	spec	Expression in plants
**AM 783**	pGGZ 2x35S::E2GFP-AtATG8I	GFP-ATG8I CDS	FastRed	spec	Expression in plants
**AM 810**	pDEST15 GST-AtATG4A WT	GST-ATG4A WT	–	amp	Expression in bacteria
**AM 811**	pDEST15 GST-AtATG4A C/A	GST-ATG4A C/A	–	amp	Expression in bacteria
**AM 812**	pDEST15 GST-AtATG4B WT	GST-ATG4B WT	–	amp	Expression in bacteria
**AM 813**	pDEST15 GST-AtATG4B C/A	GST-ATG4A C/A	–	amp	Expression in bacteria
**AM 821**	pGGC AtATG8E ΔC	AtATG8E ΔC CDS	–	amp	Entry clone for GreenGate cloning
**AM 822**	pGGC AtATG8F ΔC	AtATG8F ΔC CDS	–	amp	Entry clone for GreenGate cloning
**AM 831**	pGGZ 2x35S::E2GFP-AtATG8E ΔC	GFP-ATG8E ΔC CDS	FastRed	spec	Expression in plants

**AM 832**	pGGZ 2x35S::E2GFP-AtATG8F ΔC	GFP-ATG8F ΔC CDS	FastRed	spec	Expression in plants
**SH 117**	pGGA 2x35S	2x35S	–	amp	Entry clone for GreenGate cloning
**SH 17**	pGGZ APA1 pr FLuc-mScarlet AtATG8F/RLuc:sGFP:NLS/kanR	FLuc-mScarlet AtATG8F	Kan	spec	Expression in plants
**SH 19**	pGGZ APA1 pr FLuc-mScarlet AtATG8I/RLuc:sGFP:NLS/kanR	FLuc-mScarlet AtATG8I	Kan	spec	Expression in plants
**SH 3**	pGGC AtATG8F	AtATG8F CDS	–	amp	Entry clone for GreenGate cloning
**SH 4**	pGGC AtATG8I	AtATG8I CDS	–	amp	Entry clone for GreenGate cloning

Chlamydomonas

**Construct no.**	**Construct name**	**Backbone**	**Level in MoClo**	**Module for construction**	**Selection marker in bacteria**	**Selection marker in plants**	**Source**
**–**	pCM0-016	–	L0	–	spec	–	Crozet et al., 2018
**–**	pCM0-067	–	L0	–	spec	–	Crozet et al., 2018
**–**	pCM0-114	–	L0	–	spec	–	Crozet et al., 2018
**–**	pAGM1301	–	–	–	spec	–	Engler et al., 2014
**–**	pICH47742	–	–	–	amp	–	Engler et al., 2014
**–**	pICH47751	–	–	–	amp	–	Engler et al., 2014
**–**	pAGM8043	–	–	–	spec	–	Engler et al., 2014
**–**	pICH50892	–	–	–	amp	–	Engler et al., 2014
**pCM0-YZ77**	L0-CrATG8-gDNA-B5	pAGM1301	L0	–	spec	–	This study
**pCM0-YZ79**	L0-CrATG8ΔC-gDNA-B5	pAGM1301	L0	–	spec	–	This study
**pCM1-31**	L1-Hygro	pICH47751	L1	pICH47751, pCM0-17, pCM0-73, pCM0-115	amp	hyg	Zou et al., 2024
**pCM1-YZ103**	L1-mCherry-CrATG8	pICH47742	L1	pICH47742, pCM0-016, pCM0-067, pCM0-YZ77, pCM0-114	amp	–	This study
**pCM1-YZ105**	L1-mCherry-CrATG8ΔC	pICH47742	L1	pICH47742, pCM0-016, pCM0-067, pCM0-YZ79, pCM0-114	amp	–	This study
**pCM2-YZ119**	L2-mCherry-ATG8-Hygro	pAGM8043	L2	pAGM8043, pCM1-YZ103, pCM1-31, pICH50892	spec	hyg	This study

**pCM2-YZ121**	L2-mCherry-ATG8ΔC-Hygro	pAGM8043	L2	pAGM8043, pCM1-YZ105, pCM1-31, pICH50892	spec	hyg	This study

Table S3. Arabidopsis transgenic lines used in this study.

Transgene	Background	Comment
2x35S::E2GFP-ATG8F	WT	Good expression
2x35S::E2GFP-ATG8I	WT	Good expression
2x35S::E2GFP-ATG8F	*atg4a-2/b-2*	Good expression
2x35S::E2GFP-ATG8I	*atg4a-2/b-2*	Good expression
2x35S::E2GFP-ATG8F	*atg7-2*	Good expression
2x35S::E2GFP-ATG8I	*atg7-2*	Good expression
pUBN ubi10::EosFP-ATG8E	WT	Somatic silencing
pUBN ubi10::EosFP-ATG8E DC	WT	Good expression
pUBN ubi10::EosFP-ATG8E G/A	WT	Somatic silencing
pUBN ubi10::EosFP-ATG8E	*atg4a-2/b-2*	Somatic silencing
pUBN ubi10::EosFP-ATG8E DC	*atg4a-2/b-2*	Good expression
pUBN ubi10::EosFP-ATG8E G/A	*atg4a-2/b-2*	Somatic silencing
pUBN ubi10::EosFP-ATG8E DC	WT	Good expression
pUBN ubi10::EosFP-ATG8E DC	*atg4a-2/b-2*	Good expression
pUBN ubi10::EosFP-ATG8E DC	*atg5-1*	Ok expression
pUBN ubi10::EosFP-ATG8A gene-CFP	WT	Good expression
pUBN ubi10::EosFP-ATG8A gene-CFP	*atg4a-2*	Good expression
pUBN ubi10::EosFP-ATG8A gene-CFP	*atg4b-2*	Good expression
pUBN ubi10::EosFP-ATG8A gene-CFP	*atg4a-2/b-2*	Good expression
pMDC43 2x35S::GFP-ATG8E	WT	Somatic silencing
pMDC43 2x35S::GFP-ATG8E DC	WT	Somatic silencing
pMDC43 2x35S::GFP-ATG8E G/A	WT	Somatic silencing
pMDC43 2x35S::GFP-ATG8E	*atg4a-2/b-2*	Somatic silencing
pMDC43 2x35S::GFP-ATG8E DC	*atg4a-2/b-2*	Somatic silencing
pMDC43 2x35S::GFP-ATG8E G/A	*atg4a-2/b-2*	Somatic silencing
pUBN ubi10::EosFP-ATG8E DC	*atg4a-2*	Good expression
pUBN ubi10::EosFP-ATG8E DC	*atg4b-2*	Good expression
2x35S::E2GFP-ATG8F DC	*WT*	Good expression
2x35S::E2GFP-ATG8F DC	*atg4a-2/b-2*	Good expression
2x35S::E2GFP-ATG8E DC	*atg8s*	Good expression
2x35S::E2GFP-ATG8F DC	*atg8s*	Good expression
2x35S::E2GFP	*atg8s*	Good expression
