# Supplementary material for: ATG8 delipidation is not universally critical for autophagy in plants
Source: Nat Commun. 2025 Jan 5;16:403. doi: 10.1038/s41467-024-55754-1 (PMC11701075; doi:10.1038/s41467-024-55754-1)
Supplement: Supplementary file 3 — Description of Additional Supplementary Files [file 41467_2024_55754_MOESM3_ESM.pdf]

Description of Additional Supplementary Files

Supplementary Movie 1. Chemical inhibition of ATG4 activity with IAM and NEM does not prevent accumulation of autophagic bodies in true leaves.

Representative time-lapse scans of mesophyll cells of Arabidopsis true leaves expressing EosFP–ATG8E ΔC in the WT background. Leaves of one-month-old plants were infiltrated with MQ water containing 5 µM AZD8055 and 0.5 µM ConA additionally supplemented with 1mM IAM (Iodoacetamide) or 10 mM NEM (N-ethylmaleimide) without detaching them from plants. Leaves were imaged using CLSM 24 h after the treatment start.

Supplementary Movie 2. ATG8E ΔC complements stunted root growth phenotype of *atg4a/b* under nitrogen-depleted conditions.

Representative SPIRO time-lapse data showing seedlings on control medium (top two plates) and–N medium (bottom two plates) imaged simultaneously. Charts on the right show data from the SPIRO root growth tracking assay plotted for each seedling and combined by genotype/growth conditions: black solid lines indicate root lengths plotted vs time for each seedling, dotted black lines show the predicted root length for each analyzed group, colored area indicates standard error for the root length prediction. Autophagy-deficient genotypes (*atg4a/b* and *atg5* ATG8E ΔC) show stunted growth under –N conditions, while *atg4a/b* ATG8E ΔC seedlings have normal root elongation under the same conditions.

Supplementary Movie 3. ATG8E ΔC complements weak root growth recovery of *atg4a/b* after carbon-depletion.

Representative SPIRO time-lapse data showing seedlings on carbon-depleted plates imaged simultaneously. Seeds were allowed to germinate, and seedlings were grown under normal growth conditions, after which the lights in the growth cabinet were turned off and imaging proceeded for 4 days in the dark (carbon depletion), followed by imaging for another four days under normal growth conditions (recovery).

Roots of autophagy-deficient seedlings (*atg4a/b* and *atg5* ATG8E ΔC) do not restart growth during recovery stage, while roots of *atg4a/b* ATG8E ΔC seedlings elongate normally under the same conditions.
